# Diflunisal Targeted Delivery Systems: A Review

**DOI:** 10.3390/ma14216687

**Published:** 2021-11-06

**Authors:** Petr Snetkov, Svetlana Morozkina, Roman Olekhnovich, Mayya Uspenskaya

**Affiliations:** Center of Chemical Engineering, ITMO University, Kronverkskiy Prospekt, 49A, 197101 Saint Petersburg, Russia; Morozkina.Svetlana@gmail.com (S.M.); r.o.olekhnovich@mail.ru (R.O.); mv_uspenskaya@mail.ru (M.U.)

**Keywords:** diflunisal, drug delivery systems, amyloidosis, release profile

## Abstract

Diflunisal is a well-known drug for the treatment of rheumatoid arthritis, osteoarthritis, primary dysmenorrhea, and colon cancer. This molecule belongs to the group of nonsteroidal anti-inflammatory drugs (NSAID) and thus possesses serious side effects such as cardiovascular diseases risk development, renal injury, and hepatic reactions. The last clinical data demonstrated that diflunisal is one of the recognized drugs for the treatment of cardiac amyloidosis and possesses a survival benefit similar to that of clinically approved tafamidis. Diflunisal stabilizes the transthyretin (TTR) tetramer and prevents the misfolding of monomers and dimers from forming amyloid deposits in the heart. To avoid serious side effects of diflunisal, the various delivery systems have been developed. In the present review, attention is given to the recent development of diflunisal-loaded delivery systems, its technology, release profiles, and effectiveness.

## 1. Introduction

**Figure 1:**
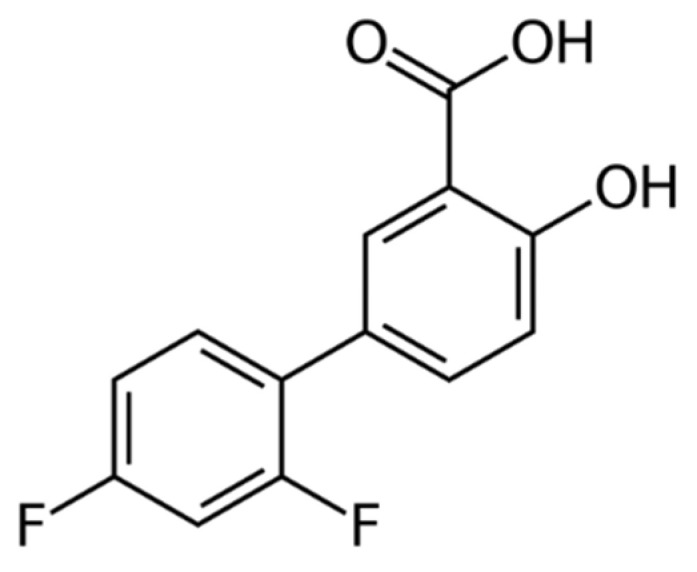
Structure of diflunisal.

Diflunisal (Figure 1) was developed by Merck Sharp and Dohme Research Laboratories in 1971 and belongs to the group of nonsteroidal anti-inflammatory drugs (NSAID) [1].

Diflunisal is mainly used for the treatment of rheumatoid arthritis, osteoarthritis, and primary dysmenorrhea. Additionally, the anticancer activity of diflunisal was proved [2].

In recent years, diflunisal received attention due to the evidence of its useful application in the treatment of cardiac amyloidosis, which was also confirmed in clinical trials [3,4,5,6,7,8]. It is the second of two small molecules investigated in animal safety studies and human clinical trials for the treatment of transthyretin (TTR) polyneuropathy [9].

In the nonrandomized study, it was demonstrated that diflunisal possesses a survival benefit similar to that of clinically approved tafamidis [10]. These data make diflunisal a perspective molecule to treat cardiac amyloidosis, because the first approved medication for the treatment of Transthyretin Amyloid Cardiomyopathy (ATTR-CM) was proposed only in 2019 [11,12].

However, long-time use of NSAIDs leads to renal dysfunction (COX-2), gastrointestinal (GI) bleeding (COX-1), and ulceration, fluid retention, and hypertension, which may result in heart failure (COX-2) in patients [13,14,15].

Furthermore, having a hydrophobic nature, diflunisal has poor water solubility. This restriction leads to unfavorable pharmacokinetic profiles and is far from the desired biodistribution [16]. Another complexity of diflunisal is its photosensitivity and the toxicity of its photoproduct. Thus, the importance of the diflunisal use and known restrictions for its application forced the development of novel delivery systems for diflunisal to reduce the side effects, increase its stability, and enhance its pharmacokinetic profile and biodistribution. 

This review covers all known data about diflunisal incorporation into different drug delivery systems. 

## 2. Drug Delivery Systems

Drug delivery systems are well-known due to their nanoparticle retention, which may be caused by the enhanced permeability and retention (EPR) effect [17]. This phenomenon provides a targeted action and allows significantly diminishing side effects, as well as decreasing the amount required for an effective dose.

The manipulation of the parameters and properties of such delivery systems opens the way to the development of new safe drug carriers with the desired drug release profile, controlled absorption, distribution, and elimination that finally improve the product’s efficacy and safety.

### 2.1. Nanoparticles

From the known nanoparticles, we pay attention to lipid-based nanostructures [18] and polymer-based nanostructures [19] due to their biocompatibility, high efficacy, versatility, and perspectivity.

Liposomes and lipid-based nanoparticles have high levels of biocompatibility and biodegradability and represent a suitable platform for modern drug delivery systems for application in medicine and bioengineering. Such systems are able to entrap both hydrophilic drugs and hydrophobic ones [18].

There are two different groups of polymeric nanoparticles: nanospheres or nanocapsules [20]. Polymeric nanospheres in general have a complete solid sphere matrix based on a polymer (biopolymer) with homogeneously dispersed drug. By contrast, polymeric spherical nanocapsules contain liquid or solid matter as a functional inner core and an external polymeric coating as a shell, preventing the burst drug release caused by various factors such as pH, temperature, biocatalysts, etc. In addition, the shell could be modified by smart (functional) molecules, which are able to interact with biological targets, leading to a variety of biological responses [21]. Multifunctional nanocapsules with layer-by-layer assembly also became widespread as modern drug delivery systems. Such technology allows obtaining pH-responsive targeted delivery systems with a high level of controlled physicochemical, biological, and therapeutic properties [22].

**Figure 2:**
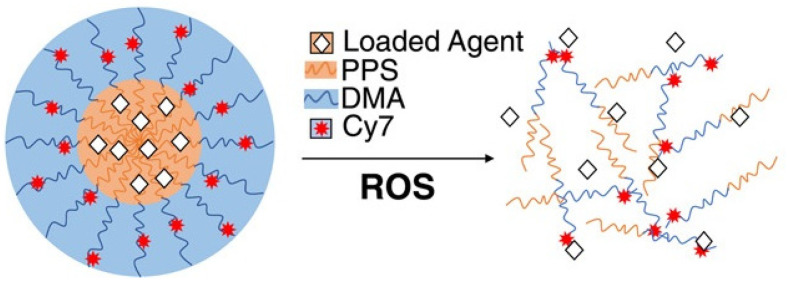
Schematic representation of poly(propylene sulfide) nanocarriers with loaded pharmaceutical agents. PPS—poly(propylene sulfide); DMA—N,N-dimethylacrylamide; Cy7—Cy7-amine, a fluorescent marker; ROS—reactive oxygen species. Reproduced from [23], with permission from John Wiley and Sons, 2021.

Thus, Caleb A. Ford et al. [23] used diflunisal-loaded nanoparticles based on poly(propylene sulfide), which were obtained by the oil-in-water emulsion method. The authors used two methods to fabricate polymer nanoparticles: a solvent evaporation technique to improve diflunisal loading parameters and a microhydrodynamics method to enhance nanoparticle product yield (for in vivo experiments). A schematic representation of poly(propylene sulfide) nanocarriers with loaded drugs is demonstrated in Figure 2.

Diflunisal-loaded nanoparticles have a mean diameter equal to 65.4 ± 0.4 nm and could be administered parenterally and, consequently, may deliver pharmaceutical agents directly to the target tissues. It was demonstrated that poly(propylene sulfide) nanocarriers are collected at the infected tissues in a murine model of post-traumatic staphylococcal osteomyelitis and allow delivering diflunisal to contaminated bone, while pure diflunisal causes bacterial colonization of the surface. Diflunisal-loaded poly(propylene sulfide) nanoparticles decrease *S. aureus*-mediated bone degradation without recidivation of the infection. 

**Figure 3:**
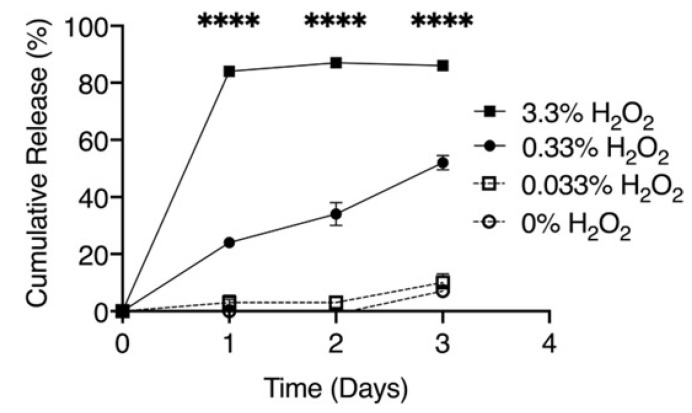
Cumulative release of diflunisal from poly(propylene sulfide) nanoparticles with different concentrations of the oxygen-derived radicals H_2_O_2_. **** *p* < 0.0001. Reproduced from [23], with permission from John Wiley and Sons, 2021.

These authors previously demonstrated [24] that poly(propylene sulfide) nanoparticles represent a reactive oxygen species-responsive drug nanocarrier for delivery of the Gli2 inhibitor, GANT58, to the targeted metastases in bone cancer. It was discussed that poly(propylene sulfide)-based biocarriers could be degraded in the presence of high levels of reactive oxygen species (ROS) and provide drug release at the target tissues and organs [23]. The cumulative release of diflunisal from poly(propylene sulfide) nanoparticles depending on different concentrations of the oxygen-derived radicals H_2_O_2_ is shown in Figure 3.

The technology of obtaining the properties of solid lipid nanoparticles for localized or skin application has been described [25]. The nanoparticles were fabricated by a hot homogenization technique based on the microemulsification method and finally coated with Carbopol 934. Compritol^®^ ATO 888 (Glyceryl dibehenate) was used as a selected lipid. The nanoparticles obtained have a spherical shape with a mean diameter equal to 124.0 ± 2.07 nm. 

**Figure 4:**
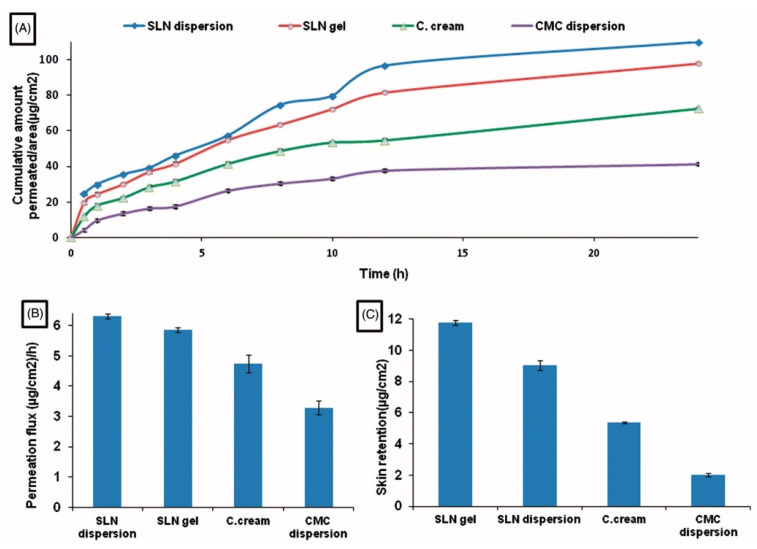
Time-dependent curve of cumulative amount permeated/area (**A**), permeation flux (µg/cm^2^/h) (**B**), and skin retention (µg/cm^2^) (**C**) of different diflunisal-loaded medical substances. SLN dispersion—solid lipid nanoparticles dispersion; SLN gel—solid lipid nanoparticles gel; C. cream—conventional oil/water (o/w) cream; CMC dispersion—aqueous diflunisal dispersion in 0.5% solution of sodium carboxymethyl cellulose (CMC). Reproduced from [25], with permission from Taylor & Francis, 2021.

A significant difference has been demonstrated between diflunisal permeation flux and skin retention from solid lipid nanoparticles’ dispersion in water and from solid lipid nanoparticles gel in comparison with permeation from diflunisal dispersion in aqueous diflunisal dispersion in 0.5% solution of sodium carboxymethyl cellulose (CMC) and from conventional oil/water (o/w) cream. The skin retention and cumulative amount permeated to area ratio also have a remarkable difference between various diflunisal-loaded medical substances. The results are shown in Figure 4. 

Note that the obtained nanoparticles have high stability and did not cause any type of histopathology. The therapeutic effectiveness of nanoparticles was proved by the reduction of granuloma tissue weight, mean fluid volume, and white blood cell count after the application of diflunisal-loaded solid lipid nanoparticles in the mice air-pouch arthritic model. Furthermore, the nanoparticles demonstrated better percentage suppression of edema in mice ear oedemata (xylene-induced) and in the rat hind paw oedemata (carrageenan-induced) models. These diflunisal delivery systems based on solid lipid nanoparticles have high efficacy with the absence of the gastrointestinal and hepatic side effects.

**Figure 5:**
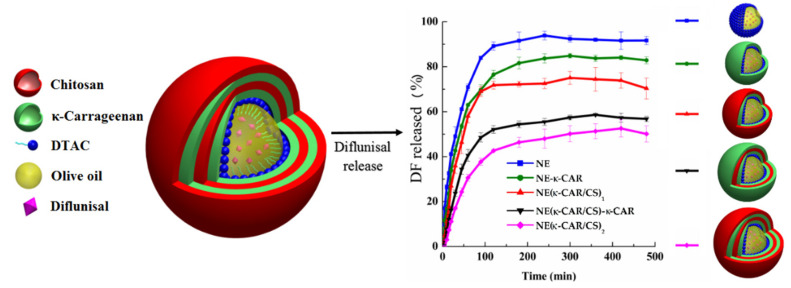
Diflunisal cumulative release profiles at pH 7.4 and 37 °C from polyelectrolyte-free nanoemulsion (NE) and nanocapsules with one (NE-k-CAR), two (NE(k-CAR/CS)_1_), three (NE(k-CAR/CS)_1_-k-CAR), and four (NE(k-CAR/CS)_2_) polyelectrolyte layers. DTAC—dodecyltrimethylammonium chloride; DF—diflunisal; CS—chitosan; k-CAR—k-carrageenan. Reproduced from [26], with permission from MDPI, 2021.

Sarai Rochín-Wong et al. [26] fabricated stable multilayer polymeric nanoparticles with a mean diameter equal to 300 nm by a layer-by-layer assembly technique. Natural polymers k-carrageenan and chitosan were used as coatings for olive oil nanoemulsion droplets. It was highlighted that the drug release profile of diflunisal is directly dependent on the number of layers. Thus, the nanoparticles with no more than two layers exhibited different transport and first-order kinetics. By contrast, nanocarriers with three and four coatings demonstrated a Case II diflunisal transport mechanism and zero-order type of kinetics, which is the main principle of the technologies for the manufacturing of pharmaceutical drugs with prolonged action [27]. Diflunisal cumulative release profiles at pH 7.4 and 37 °C from nanoemulsion (NE) and nanocapsules with various polymer layers are demonstrated in Figure 5.

Apart from polymeric systems, there are several inorganic nanoparticles, which could be used as challenging drug delivery systems: calcium phosphate nanoparticles, mesoporous silica nanoparticles, carbon nanostructures (nanotubes, nanospheres), iron oxide nanoparticles, etc. The mesoporous silica nanoparticles (MSN) attracted attention due to their tunable size, enormous surface area, porosity, possibility of surface functionalization, and biocompatibility [28].

Gennara Cavallaro et al. [29] demonstrated an example of a mesoporous silicate matrix (MSM) for the drug delivery of nonsteroidal anti-inflammatory drugs, including diflunisal. It was shown that the MSNs are able to trap drugs during soaking with subsequent release under environmental conditions equal to biological fluids. Moreover, the high affinity of such nanoparticles to aqueous solutions potentially leads to their high biocompatibility. Diflunisal-loaded system released just 20% of the drug content in the stomach. Thus, the side effects related to the oral administration of NSAID could be decreased and the targeted release of the pharmaceutical agent may be realized in the intestinal tract. 

### 2.2. Hydrogels and Oleogels

Hydrogels are defined as triaxial, cross-linked networks based on hydrophilic polymers (biopolymers) [30]. These materials attracted attention due to their unique physicochemical, physicomechanical, and biological properties, such as swelling in water or physiological environments, pH and thermal sensing, or susceptibility to other exposure, such as ions or glucose. Hydrogels as biocompatible and biodegradable materials can protect drugs, for example, peptides and proteins, from in vivo aggressive medium, which allows delivering pharmaceutical agents directly to the target tissue practically without loss of drug [31]. 

Hydrogels could be mainly divided into two groups: (a) by the type of hydrogel formation: physical, covalent, nanoparticle-containing hydrogels, and hydrogels containing cyclodextrins [32], and (b) by drug–polymer interactions: covalent conjugation, electrostatic interactions, and hydrophobic associations [33]. Different types of hydrogel formation and drug–polymer interactions allow fabricating drug delivery systems with various release mechanisms and kinetics, which is nowadays a crucial task for interdisciplinary fields such as drug development, medicine, bioengineering, and polymer chemistry.

Lipogels (oleogels, organogels) are gels involving oils or apolar liquids as a dispersing agent. More specifically, organogels represent an organic fluid that is trapped in a three-dimensional network based on thermo-reversible gel. Lecithin organogels as a particular case of lipogels based on phospholipids from egg yolks could be characterized as stable, viscoelastic, biodegradable, bio-friendly, and isotropic gels [34]. 

María Dolores Figueroa-Pizano et al. [35] developed chitosan–poly(vinyl alcohol) (PVA) hydrogels without toxic cross-linkers via the freeze–thawing method. The influence of freezing and the number of freeze–thawing cycles on the morphological and physicochemical properties of the hydrogels obtained as well as on diflunisal loading and its release have been evaluated. 

**Figure 6:**
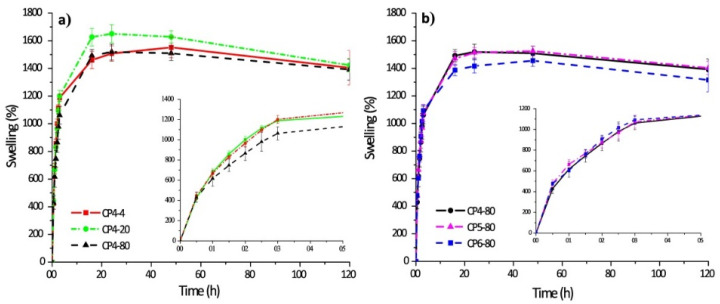
Swelling behavior of polymer hydrogels: chitosan–PVA hydrogels prepared with four cycles of freeze–thawing (**a**); chitosan–PVA hydrogels obtained at −80 °C with different numbers of freeze–thawing (**b**). In the sample abbreviation, the first number after “CP” indicates the quantity of freeze–thawing cycles, the second one indicates the temperature of freezing. Reproduced from [35], with permission from JoVE, 2021.

The swelling kinetics of chitosan–PVA hydrogels prepared with four cycles of freeze–thawing and other ones obtained at −80 °C with different numbers of freeze–thawing cycles are shown in Figure 6.

Chitosan–PVA hydrogels have a fast absorption rate of water. Thus, during the first five hours, the hydrogel samples swelled 10-fold of their initial weight, and after 20 h, the hydrogel samples swelled up to 15-fold in comparison to their initial weight (it is also an equilibrium). There are interesting notes related to each case. Thus, if the number of freezing cycles was constant, the sample CP4-80 demonstrated a lower level of swellability in the initial stage. By contrast, there are no significant differences of swelling capacity for the samples obtained at various numbers of freezing cycles. 

**Figure 7:**
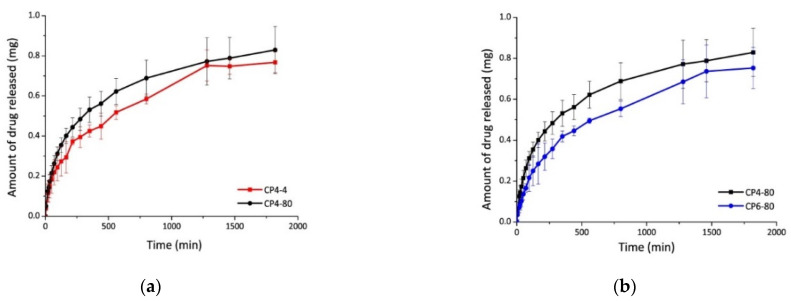
Diflunisal release profiles for hydrogels CP4-4 and CP4-80 (**a**); and CP4-80 and CP6-80 (**b**). In the sample abbreviation, the first number after “CP” indicates the quantity of freeze–thawing cycles, and the second one indicates the temperature of freezing. Reproduced from [35], with permission from JoVE, 2021.

The diflunisal encapsulation efficiency in the obtained hydrogels was equal to 70% in all cases. The release kinetics of diflunisal from the chitosan–PVA hydrogels were equal to 30 h for all samples. The sample CP4-80 hydrogel demonstrates the faster diflunisal release profile (Figure 7). The authors explain this fact by the higher porous structure of CP4-80 hydrogel as compared to the other two hydrogel samples. At the same time, there are no differences between CP4-80 and CP6-80 samples (Figure 7). Note that a burst effect was not detected for any type of the obtained hydrogels, which is important for further biomedical applications. 

The skin penetration of diflunisal from lipogel form and hydrogel microemulsion form has been investigated and compared [36]. Organogel was prepared from a composition consisting of 50% soya bean lecithin (two types), 27% butyl lactate, and 23% water by disintegration of the lecithin in butyl lactate following water addition. Diflunisal was added in the lecithin/butyl lactate mixture before adding water. By contrast, microemulsion-based gel was obtained by the mixing of butyl lactate, water, and surfactant mixture [37]. This gel has better spreadability and demonstrates a 5.07-fold increase in the transdermal flux as it was compared to Carbomer^®^ 934 gel. In the experiments in mice, gel significantly reduces the licking time as compared to the control group. 

**Figure 8:**
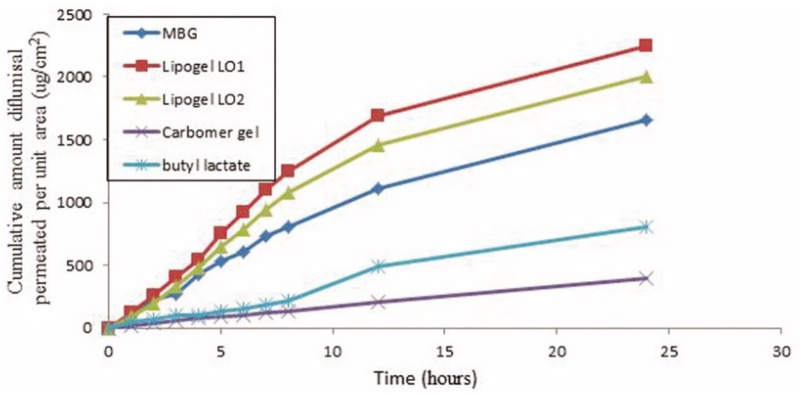
Human skin penetration profiles of diflunisal from microemulsion-based hydrogel, organogels (LO1 and LO2), carbomer gel, and butyl lactate into phosphate-buffered saline (PBS) (pH 7.4) at 32 °C. MBG—microemulsion-based hydrogel form; LO1—organogel based on Lipoid S75; LO2—organogel based on Phospholipoin 85 G. Reproduced from [36], with permission from Taylor & Francis, 2021.

**Figure 9:**
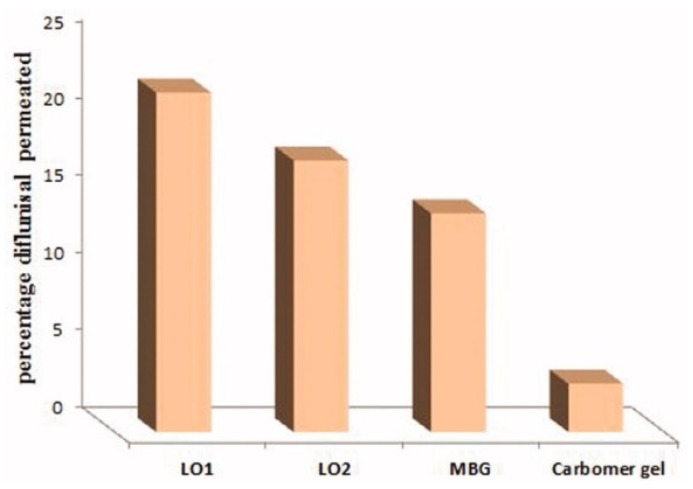
Percentage diflunisal permeated through human skin from lipogels (LO1 and LO2), microemulsion-based hydrogel (MBG), and carbomer gel after 24 h. MBG—microemulsion-based hydrogel form; LO1—organogel based on Lipoid S75; LO2—organogel based on Phospholipoin 85 G. Reproduced from [36], with permission from Taylor & Francis, 2021.

The cumulative amount of diflunisal permeated through human skin from the organogels obtained (type LO1 based on Lipoid S75 and LO2 based on Phospholipoin 85 G) and from microemulsion-based hydrogel form (MBG), carbomer gel, and butyl lactate is demonstrated in Figure 8. A continuous growth of diflunisal in the receptor chambers as time passed was detected for all compared compositions. Lipogel LO1 demonstrated the ultimate permeability level (210.8 µg cm^−2^ h^−1^) and advanced percentage diflunisal permeation (Figure 9).

pH-Sensitive hydrogels based on bovine serum albumin hydrophilic microspheres were obtained by Francesca Iemma et al. [38]. The results demonstrated that the drug release profile depends on diflunisal–polymer matrix interaction and diffusional restriction related to the degree of crosslinking in the microparticles obtained. At pH 1.0, extremely low diflunisal quantity was detected (*w*/*w* < 10% after 2 h). When the pH rose to 6.8, the diflunisal released amount increased (*w*/*w* > 75% after 24 h).

### 2.3. Complexes

One of the most interesting and investigated drug delivery systems is complexes based on cyclodextrins. Cyclodextrins and their derivatives represent multipurpose oligosaccharides with unique physicochemical, chemical, and biological properties, and in general, they are used as multifunctional agents to enhance the stability, aqueous solubility, dissolution velocity, and biological availability of hydrophobic pharmaceutical agents [39]. Due to the chemical structure of the cyclodextrins, individual hydrophobic drugs or hydrophobic chains of massive pharmaceutical agents are entrapped into water-soluble inclusion complexes [40] without the changing of drug characteristics [41]. Formed water-soluble inclusion complexes are known to be targeted delivery systems due to their biocompatibility, low toxicity, degradability, and self-assembling potency. The structure of cyclodextrins complexes with drugs resemble nanocarriers such as nanoparticles, nanospheres, nanomicelles, etc. At the same time, pharmaceutical agents with different nature (hydrophilic or hydrophobic) could be incorporated into water-soluble or insoluble parts of cyclodextrins inclusion complexes [41]. 

The first example of the inclusion complexes of diflunisal with cyclodextrins was described in 1987 and further studied by UV-visible and ^19^F nuclear magnetic resonance spectroscopic methods [42,43,44]. It was determined that the formation of the inclusion complexes with β-cyclodextrin (βCD) in the ratios 1:1 and 1:2 includes two equilibria. In the case of α-cyclodextrin (αCD), only the diflunisal·αCD complex was detected.

In the next paper [45], β-cyclodextrin (βCD), γ-cyclodextrin (γCD), and hydroxypropyl-β-cyclodextrin (HPβCD) were used to form complexes with diflunisal (phosphate buffer, pH 7.4, at 5–37 °C). 

Cyclodextrin complexes of diflunisal based on β-cyclodextrin or hydroxypropyl β-cyclodextrin were obtained by Mehreen Bashir et al. [46]. The different hydrophilic polymers (carboxymethyl cellulose sodium, polyvinyl alcohol, and poloxamer-188 (PXM188)) were used, and the influence of polymer addition on the solubility and dissolution of diflunisal entrapped into cyclodextrin complexes has been compared.

The complexes containing diflunisal with β-cyclodextrin or hydroxypropyl β-cyclodextrin were prepared with weight ratios equal to 1:1, 1:2, and 1:4, in distilled water and methanol (1:1). In the case of ternary complexes with hydrophilic polymers, the latter were individually added to binary cyclodextrin complexes with diflunisal and β-cyclodextrin or hydroxypropyl at a 1:2 volume ratio. 

Solubility data of diflunisal and complexes with β-cyclodextrin or hydroxypropyl β-cyclodextrin are presented in Table 1. 

The solubility of diflunisal in distilled water (30 °C, 72 h) was improved after the incorporation into βCD and HPβCD inclusion complexes. For complexes with a drug ratio to cyclodextrin equal to 1:2, the aqueous solubility is at a high level (increasing from 44.6 ± 0.02 for pure drug to 450.3 ± 0.6 µg/mL for β-cyclodextrin and to 907.5 ± 0.5 µg/mL for hydroxypropyl β-cyclodextrin complexes); for this reason, the authors chose this ratio for the further investigations. 

When the hydrophilic polymers were added, the water solubility of diflunisal was increased. The highest solubility of diflunisal was observed for the cyclodextrin complex with PXM-188. A lower solubility of diflunisal was achieved with PVA addition, and the lowest solubility was achieved with CMC-Na. The maximum of diflunisal solubility (1259.5 ± 0.5 µg/mL) was detected for the complex with hydroxypropyl β-cyclodextrin and Poloxamer-188. This could be explained by the crystalline structure and amorphous form of such cyclodextrin complexes [46].

**Figure 10:**
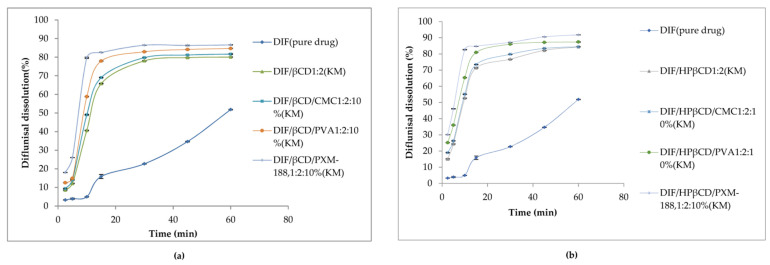
Dissolution profiles of diflunisal, and cyclodextrin inclusion complexes with β-cyclodextrin (**a**) and hydroxypropyl β-cyclodextrin (**b**). DIF—diflunisal; β-CD—β-cyclodextrin; HPβ-CD—hydroxypropyl β-cyclodextrin; CMC—carboxymethyl cellulose sodium; PVA—polyvinyl alcohol; PXM-188—poloxamer-188. Reproduced from [46], with permission from MDPI, 2021.

Dissolution profiles of diflunisal and developed cyclodextrin complexes with various contents of additional polymers in distilled water at 37.0 ± 0.5 °C are demonstrated in Figure 10. Evidently, diflunisal as a hydrophobic agent has the worst solubility. The diflunisal release from β-cyclodextrin and hydroxypropyl β-cyclodextrin complexes was higher in comparison with pure diflunisal by 11–21-fold. Hydrophilic polymers allow increasing the release rate of diflunisal by 15–28-fold. The complex of hydroxypropyl β-cyclodextrin and Poloxamer-188 demonstrated the highest diflunisal solubility: 1259.5 ± 0.5 μg/mL. The complexes with polyvinyl alcohol and carboxymethyl cellulose (CMC) also demonstrated the enhanced water dissolution rate of diflunisal in comparison with complexes without additional polymers.

In addition to cyclodextrin inclusion complexes, there are complexes with block copolymers of poly(ethylene glycol) (PEG) and poly(ε-caprolactone) (PCL) [47].

Metal–organic complexes represent the novel type of advanced and topical materials which could be obtained by the self-aggregation of metal ions (as well as clusters) and polydentate ligands [48]. A metal–organic complex consists of transition metal or lanthanide ions (or clusters) and the surrounding organic ligands; thus, the structure of such a complex represents the linking hub (core) with a spreader bar (strut). Due to its unique structure, optimal and controllable porosity, extra-high surface area, and ability of chemical modification, metal–organic complexes are very good candidates for modern drug delivery vehicles [49]. However, such complexes in spite of their potential efficiency usually contain harmful metals or incompatible organic ligands. Known methods for the development of drug delivery systems (for instance, for tumor therapy) are always related to the imbalance of coordination linkage in acidic media (which hinders oral drug delivery via the gastrointestinal tract), or complicated by the following modification, which could lead to the alteration of an entrapped pharmaceutical agent [50].

**Figure 11:**
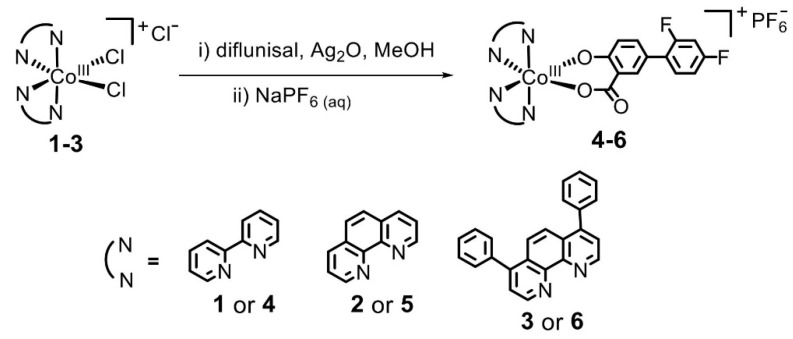
Mechanism of formation of the cobalt(III)–polypyridyl complexes with diflunisal. Reproduced from [51], with permission from the Royal Society of Chemistry, 2021.

David O. Abe et al. [51] obtained a set of diflunisal-loaded cobalt(III)–polypyridyl complexes (Figure 11), which possess the ability to differentially release the drug under acidic conditions.

The authors highlighted that these complexes are able to kill the cancer stem cells and cancer cells even at low concentrations. The antitumor effect of complexes could be characterized as the targeted and selective mode against cancer cells, while normal cells are untouched. It is important that the killing mechanism of cancer cells by one of composition (number 5) includes the DNA damage and inhibition of COX-2. This study demonstrates that metal–organic complexes based on cobalt (III) possess the reduction-activated nature and could be used as potential anticancer drug delivery systems without toxicity against normal cells.

**Figure 12:**
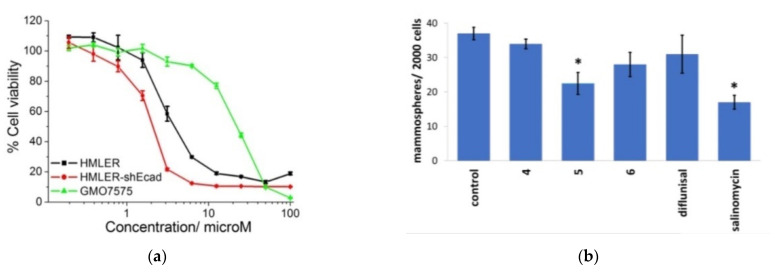
Dose–response relationship of cobalt (III) complex (composition number 5) against HMLER (bulk breast cancer cells), HMLER-shEcad (breast cancer stem cells), and GMO7575 (skin fibroblast) cell lines after 72 h of incubation (**a**). Quantification of mammosphere morphosis with HMLER-shEcad cells without and with the addition of complexes obtained, pure diflunisal, and pure salinomycin at their respective IC_20_ values for 5 days (**b**). Error bars are equal to standard deviation (SD); for Student’s *t*-test * *p* < 0.05. Reproduced from [51], with permission from the Royal Society of Chemistry, 2021.

The results of cytotoxicity analysis of composition number 5 against breast cancer cells HMLER and breast cancer stem cells-enriched HMLER-shEcad demonstrates that the complexes have high potential as the effective therapeutic treatment with cancer stem cells’ selective ability in contrast to cancer and normal cells. The diflunisal complexes did not possess any toxicity toward normal skin fibroblast cells (line GM07575), which confirms the targeted effect of the compositions obtained. Dose–response curves of the cobalt (III) complex (composition number 5) toward HMLER, HMLER-shEcad, and GMO7575 cell lines after 72 h of incubation and quantitative data of mammosphere morphosis with HMLER-shEcad cells with and without the addition of cobalt (III) complexes, pure diflunisal, and pure salinomycin at their respective IC_20_ values for 5 days are demonstrated in Figure 12a,b, respectively.

**Figure 13:**
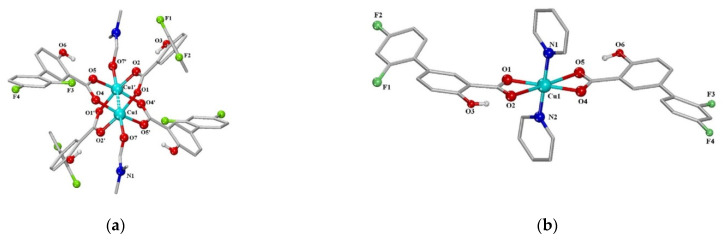
A schematic representation of the molecular structure of Cu (II) complexes: diflunisal with N,N-dimethylformamide (**a**); diflunisal with pyridine (**b**). Reproduced from [52], with permission from Elsevier, 2021.

The next series of neutral metal–organic diflunisal-loaded copper (II) complexes with the presence of the O-donor ligand *N*,*N*-dimethylformamide or N-donor heterocyclic ligands (2,2′-bipyridine, 2,2′-bipyridylamine, 1,10-phenanthroline and pyridine) were obtained and characterized by Stella Fountoulaki et al. [52]. The structures of the Cu (II) complexes of diflunisal with *N*,*N*-dimethylformamide and pyridine are depicted in Figure 13a,b, respectively.

The structures of complexes were confirmed by X-ray data. It was demonstrated that diflunisal and its complexes have good binding ability to bovine and human serum as well as to calf-thymus DNA. Such complexes may delivery metal ions and complexes with active agents through the bloodstream directly to targeted tissue, organs, or systems. 

### 2.4. Co-Crystals

Pharmaceutical co-crystals are a member of a co-crystals group in which one ingredient represents a medicament molecule (or a biologically active agent) and the second is a safe food or pharmaceutical additive. The two ingredients are bonded by a hydrogen link in an adjusted stoichiometric proportion in the crystalline grid. Most recently, pharmaceutical co-crystals have shown significant potential to change and regulate the physical, physicochemical, and pharmacokinetic characteristics of active pharmaceutical ingredient: for example, the solubility and dissolution velocity, melting point, stability, permeability, biological availability, etc. [53,54,55,56].

In the co-crystals, the drug solubility could be increased by four to 20 times in comparison with pure drugs. It is important that the co-crystals do not change the therapeutic properties of the pure pharmaceutical agent, which is important for modern drug delivery systems and personalized medicine [56].

Évora et al. [57] demonstrated the first example of diflunisal co-crystal with antituberculosis pharmaceutical agent pyrazinamide. The synthesis of co-crystal was produced by three various methods: annealing a grinded equimolecular mixture at 80 °C, annealing of mixture with a low degree of crystallinity at the ambient temperature, and ball-mill mixing carried out with ethanol. Only physicochemical characteristics of co-crystal obtained have been evaluated. Nevertheless, it was highlighted that the co-crystals developed have high potential to improve the solubility of diflunisal in aqueous and physiological liquids and, consequently, improve the bioavailability, especially in combinational drug therapy.

**Figure 14:**
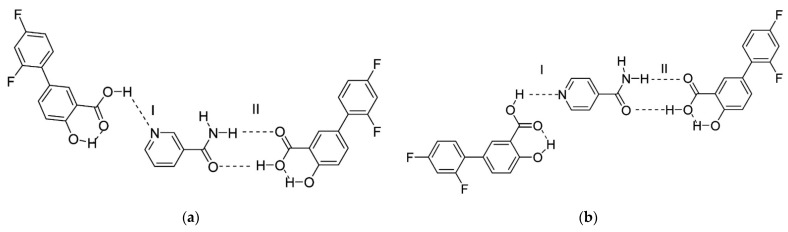
Structure of co-crystals formed between diflunisal and (**a**) nicotinamide, (**b**) isonicotinamide. Here, (I) represents the carboxylic acid–pyridine synthon; (II) represents the carboxylic acid–amide synthon. Reproduced from [58], with permission from American Chemical Society, 2021.

The next article about co-crystals of diflunisal with nicotinamide and isonicotinamide was published in 2013 [58]. The pyridine-carboxylic acid hetero synthon and amide-carboxylic acid hetero synthon are obtained via the formation of co-crystals. The structures of co-crystals investigated are shown in Figure 14. Such co-crystals demonstrated an enhanced dissolution rate and higher water solubility in comparison with the native diflunisal.

**Figure 15:**
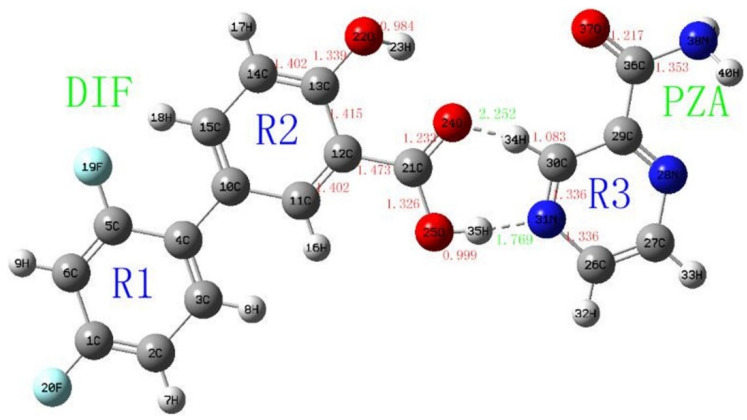
Structure of co-crystal formed between diflunisal (DIF) and pyrazinamide (PZA) and bond lengths (Å). Reproduced from [59], with permission from Elsevier, 2021.

The next study [59] continued the investigations of diflunisal–pyrazinamide co-crystal by terahertz spectral characterization and analysis by normal Raman spectroscopic methods. Theoretical data and results of the terahertz spectral analysis of the co-crystal obtained demonstrate that the diflunisal is bound with pyrazinamide by the carboxylic acid–pyridine hetero-synthon linking (see Figure 15). 

**Figure 16:**
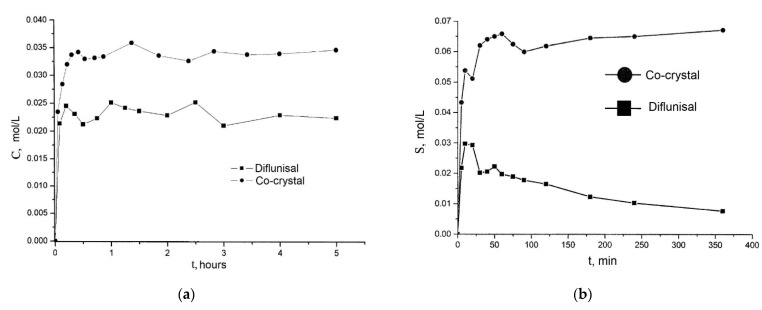
The release profiles of (**a**) native diflunisal (■) and from diflunisal–theophylline co-crystal (●); (**b**) native diflunisal (■) and from diflunisal–isoniazid co-crystal. Adapted from [60,61], 2021.

Co-crystals of diflunisal with theophylline were patented in 2015 [60]. The molar ratio of components obtained was equal to 1:1. It was demonstrated that the diflunisal–theophylline co-crystal allows enhancing drug solubility in water at the room temperature by 1.6 times (see Figure 16a). Co-crystals of diflunisal with isoniazid having the molar ratio of 1:1 were also patented [61]. The increased solubility in water by five times in comparison to pure diflunisal is reported (see Figure 16b). 

### 2.5. Solid Dispersions

Solid dispersions belong to the group of the very effective and useful techniques for the improvement of the drug release kinetics. Solid dispersion represents the solid phase where the active agent is scattered into another substance (compound) [62]. 

There are several classification ways of solid dispersions. Firstly, solid dispersions could be divided into solid solutions and eutectic mixtures in relation to the molecular arrangement. Moreover, there are three generations of solid dispersions: the first generation represents the solid dispersions, which are obtained based on crystalline carriers; the second generation demonstrates the solid dispersions based on amorphous carriers; and the solid dispersions of the third generation consist of a surfactant carrier or a blend of surfactants and amorphous polymers [63].

**Figure 17:**
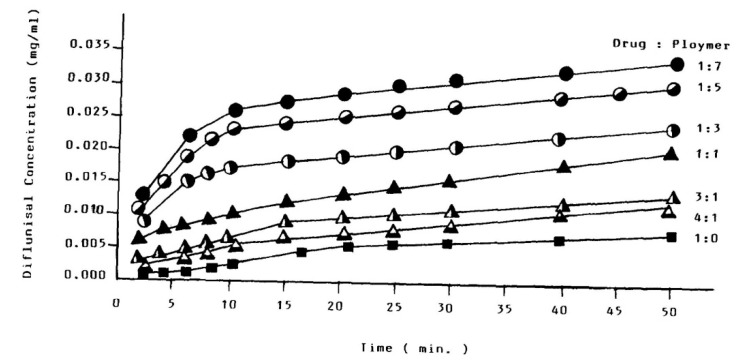
The diflunisal release profiles from PEG solid dispersions of different drug/polymer ratios. Reproduced from [64], with permission from Elsevier, 2021.

The increased solubility of diflunisal when dispersed in PEG was initially detected by N.M. Najib et al. [64]. The diflunisal release profiles from various compositions are demonstrated in Figure 17. There is no chemical interaction between diflunisal and PEG neither in solution nor in the solid state. It was reasoned that the increase in diflunisal solubility could be caused by several origins, such as a local solubilizing effect, particle size reduction of the diflunisal grains, changes in the surface properties of drug particles, etc.

Solid dispersions of diflunisal and polyvinylpyrrolidone (PVP) were prepared by the solvent method. The proportions of polymer and drug were from 20:80 to 50:50 [65]. The higher dissolution rates of diflunisal from solid-state dispersion in comparison with the physical mixture were demonstrated.

The photoprotective strategy for diflunisal was proposed by the formation of solid dispersions with Eudragit RS100 and RL100 with different polymer–drug ratios [66]. The dispersion of the diflunisal in the polymer matrix changes its release profile, leading to a slow and prolonged kinetic profile. Furthermore, in vitro assays demonstrate the alteration of the susceptibility of the diflunisal photochemical sensitization toward the cellular membrane in the presence of a polymer matrix. 

E.E. Zein El Din et al. [67] obtained diflunisal-containing solid dispersions based on Eudragit RS100, S100, L100, and ethyl cellulose. Interestingly, the formulations have different sensibility and release profiles: related to pH, time-related, and pH–time-dependent. Such systems could be used for the colon-specific delivery of diflunisal for the treatment of variety diseases, for example, nonspecific ulcerative colitis, cirrhosis disease, intestinal amoebiasis, and colon tumor. It was shown that the coupling of pH- and time-dependent profiles have better properties than systems with individual dependency. 

### 2.6. Dendrimers

**Figure 18:**
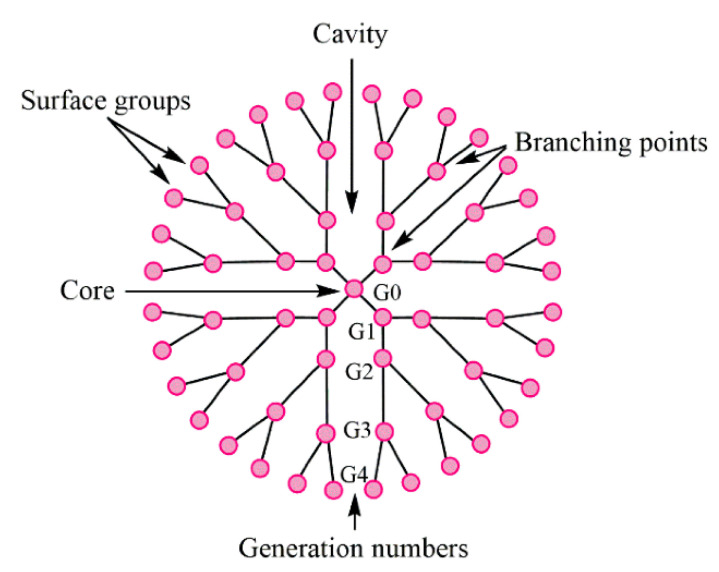
The structure of typical dendrimer. Reproduced from [68], with permission from MDPI, 2021.

Dendrimers represent synthetic polymers with branched structures of repeating units. The branches start from the core and could have anionic, neutral, or cationic outward functional groups, which provide a hydrophobic or hydrophilic property to such a branched structure. Dendrimers belong to nanoscale molecules with actinomorphous, globular, spherical-shaped, three-dimensional, isodispersed, and homogenous structures [68,69]. The nanomolecules having the diameters from 1 to 15 nm [70] are characterized by the number of generations they consist of. Nowadays, it is known to have 13 generations (G0–G12); the number demonstrates the order of the branch [68]. The layout view of the dendrimer structure is depicted in Figure 18.

**Figure 19:**
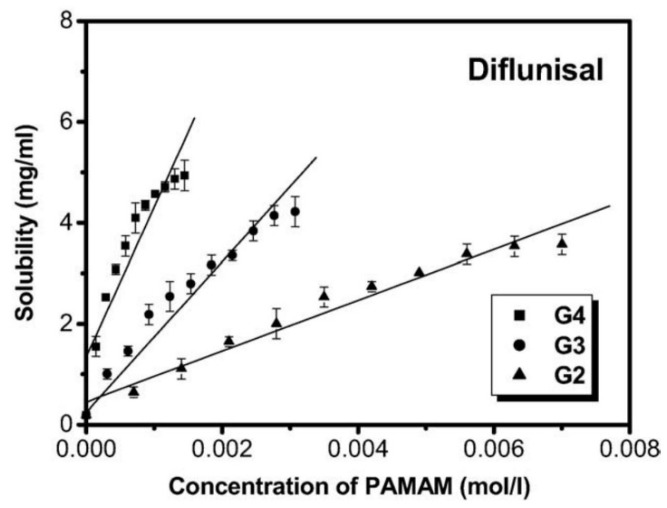
Solubility of diflunisal in the presence of dendrimers. Reproduced from [71], with permission from Elsevier, 2021.

Cheng Yiyun et al. [71] used the ethylenediamine (EDA) core polyamidoamine (PAMAM) dendrimers and demonstrated its efficacy for the improvement of the diflunisal solubility. The authors obtained dendrimers with a variety of generations (G2, G3, G4) and different PAMAM concentration (see Figure 19). The solubility of diflunisal in the dendrimer solutions was proportional to PAMAM concentration; the drug solubility in G4 solutions was higher than solubility in G2 and G3, which could be related to the hydrogen bonding between tertiary amines being in dendrimer internal cavities and the drug molecules, and it could also be related to the electrostatic interaction of external amine groups of the dendrimer and the carboxyl groups of the diflunisal [71].

**Figure 20:**
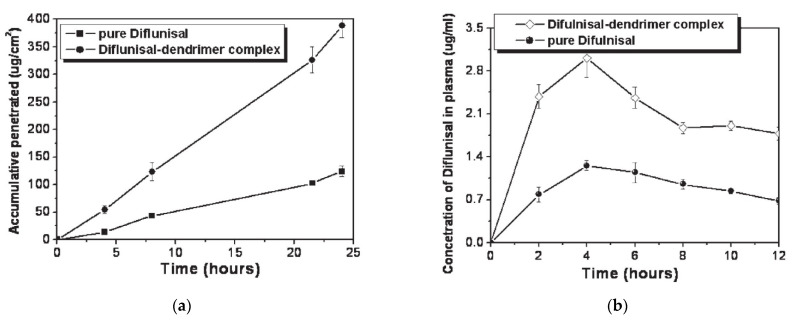
(**a**) Accumulative amount of diflunisal permeated through rat skin from pure diflunisal dispersed in saline solution (■) and aqueous solution of a drug–dendrimer complex (●); (**b**) diflunisal concentration in animal plasma after treatment of pure diflunisal (●) and diflunisal–dendrimer complexes (◇). Reproduced from [72], with permission from Elsevier, 2021.

The ability of PAMAM dendrimers (generations G1–G5) to improve the transdermal delivery of diflunisal and its pharmacokinetic and pharmacodynamics profiles were analyzed [72]. A cumulative amount of drug permeated through rat skin from diflunisal suspended with concentration equal to 0.002 g/mL in saline solution and a diflunisal–dendrimer complex dissolved in deionized water and diflunisal concentration in animal plasma are demonstrated in Figure 20.

The results of both in vitro and in vivo studies demonstrates that polyamidoamine dendrimers allow enhancing the transdermal delivery of diflunisal. The cumulative permeated amount of diflunisal across the dissected rat skin was ultimately enhanced due to the use of diflunisal–dendrimer complexes in comparison with diflunisal suspension. The transdermal drug bioavailability was higher in the case of diflunisal–dendrimer complexes. Blood drug level analysis has shown that diflunisal–PAMAM complexes lead to a 2.48-fold increase in drug level. 

The efficacy of polypropylene oxide cored polyamidoamine (PPO*PAMAM) dendrimers was demonstrated [73]. The influence of dendrimer concentration, generation, and size of the core on the diflunisal solubility was analyzed. The results demonstrated the approximately proportional dependence of drug solubility from dendrimer concentration and its generation.

**Figure 21:**
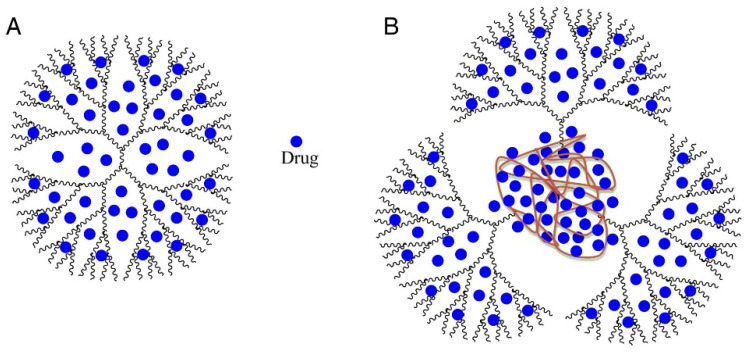
The probable structure of drug-loaded PAMAM dendrimer (**A**); and drug-loaded PPO*PAMAM dendrimer (B). PAMAM—polyamidoamine; PPO*PAMAM—polypropylene oxide cored polyamidoamine. Reproduced from [73], with permission from Elsevier, 2021.

The solubility improvement ability of PPO*PAMAM dendrimers are four-fold higher than the ethylenediamine-cored PAMAM ones. It is known that PAMAM dendrimers have many non-polar cavities (“pockets”), which could be loaded with drugs or other guest molecules having molecular weights (MW) up to 0.8 kDa [73]. Interestingly, PPO*PAMAM dendrimers possess large polypropylene oxide “pockets” in the core. For this reason, such dendrimers have better solubility improvement ability than EDA-cored polyamidoamine dendrimers. The probable host–guest structure of dendrimers and loaded drugs is demonstrated in Figure 21. 

## 3. Conclusions and Future Perspectives

Well-known NSAID diflunisal, being clinically used for a long time to treat inflammatory diseases, possesses serious side effects that restrict its application. The recent data of clinical trials reveal that diflunisal stabilizes the transthyretin tetramer and may prevent misfolding monomers and dimers from forming amyloid deposits in the heart. These data attract new interest in diflunisal as a reposited drug for the treatment of cardiomyopathies.

The serious side effects, low water solubility and bioavailability, and photosensitivity of diflunisal may be overcome by the development of new drug delivery systems. 

This review highlighted the known available data about such systems, which may be divided into main five groups: hydrogels and oleogels, complexes, co-crystals, solid dispersions, and dendrimers. Although metal–organic complexes and the co-crystals do not directly belong to the drug delivery family, using them allows organizing the targeted delivery of improved drugs, and the data about such structures give important information about the interactions in the structures formed. 

Most investigations missed many quite important evaluations, including the lack of the data on in vitro and in vivo models, which makes it difficult to draw any conclusions about the correlation between the structure of such complexes and biological activity. 

Accordingly, for the data summarized in the Table 2, the following may be concluded:

(1) No burst release effect was detected in all developed systems, which underlines the importance of drug delivery systems for diflunisal for prolonged use;
(2) Most systems demonstrated a selective mode of action both in in vitro and in vivo studies;
(3) Cyclodextrin and hydroxypropyl-β-cyclodextrin complexes as well as dendrimers and nanoparticles are the most effective drug delivery systems for diflunisal;

Additionally, the development of new delivery systems based on natural polymers such as hyaluronic acid, chitosan, etc. are missing up to date and may open new strategic ways for the discovery of safe and effective drug systems with controlled properties.

## Figures and Tables

**Table 1:** Solubility data of diflunisal and various cyclodextrin inclusion complexes. Reproduced from [46], with permission from MDPI, 2021.

DIF:CD (*w*/*w*)	Solubility (µg/mL)	
	βCD	HPβCD
1:0	44.6 ± 0.02	44.6 ± 0.02
1:1	284.5 ± 0.5	765.5 ± 0.5
1:2	450.3 ± 0.6	907.5 ± 0.5
1:4	500.5 ± 0.5	940.4 ± 0.5
1:2 (2.5% PVA)	789.6 ± 0.5	965.5 ± 0.5
1:2 (5.0% PVA)	846.3 ± 0.6	1049.3 ± 0.6
1:2 (10.0% PVA)	904.1 ± 0.5	1190.3 ± 0.6
1:2 (2.5% CMC-Na)	692.1 ± 0.3	924.3 ± 0.6
1:2 (5.0% CMC-Na)	723.6 ± 0.6	1000.5 ± 0.5
1:2 (10.0% CMC-Na)	800.6 ± 0.5	1089.6 ± 0.6
1:2 (2.5% PXM-188)	791.2 ± 0.7	998.5 ± 0.5
1:2 (5.0% PXM-188)	894.4 ± 0.5	1181.6 ± 0.6
1:2 (10.0% PXM-188)	930.0 ± 0.5	1259.5 ± 0.5

Abbreviations: DIF—diflunisal. CD—cyclodextrins (βCD—β-cyclodextrin; HPβCD—hydroxypropyl β-cyclodextrin). PVA—polyvinyl alcohol. CMC_Na—carboxymethyl cellulose sodium. PXM-188—Poloxamer-188.

**Table 2:** Summarized data.

№	System; Method; Size Obtained	Cell Line/In Vitro/In Vivo Models; Dose	Diflunisal Release, Biodistribution	Refs.
1	Poly(propylene sulfide; oil-in-water emulsion method; 65.4 ± 0.4 nm	10 μg/mL, parenterally; -murine preosteoblast MC3T3-E1 subclone 4 cell line; -colony of *S. aureus* from a tryptic soy agar; -inhibits the cytotoxicity of *S. aureus* supernatants; -decreases *S. aureus*-induced cortical bone loss during osteomyelitis (on Day 14); -had no effect on bacterial burdens.	-maximum release is reached at 33% of H_2_O_2_ at 24 h; -biodistribution (FVB/NJ mice with osteomyelitis of livers, kidneys, and spleens) up to 24 h post injection.	[23]
2	Carbopol 934, Glyceryl dibehenate (Compritol^®^ ATO 888); microemulsification method; 124.0 ± 2.07 nm	-mice air pouch model; -in vivo pharmacodynamic studies; -better percentage suppression of oedema in mice ear oedema model (xylene induced) and rat hind paw oedema (carrageenan induced); -mean leukocyte count was reduced to 4500 ± 436 cells/mm^3^ in SLN gel from 173 800 ± 1950 cells/mm^3^ in positive control; -gastrointestinal and hepatic side effects were avoided; -anti-inflammatory efficacy of DIF SLN gel as compared with conventional cream; -did not cause any type of histopathology.	-permeation flux was maximum for solid lipid nanoparticles dispersion; -skin retention was maximum for solid lipid nanoparticles gel; -high-efficacy therapeutic effects were observed at a much less reduced dose as compared with conventional oral dose.	[25]
3	k-Carrageenan and chitosan; layer-by-layer assembly technique; 300 nm	-nanocarriers with three and four coatings demonstrated Case II diflunisal transport mechanism and zero-order type of kinetics;	-the release profile is directly dependent on the number of layers; -maximum of cumulative release was reached at 100 min for all compositions with maximum as 95% for nanoemulsion and minimum as 45% for four (NE(k-CAR/CS)2) polyelectrolyte layers.	[26]
4	Chitosan–poly(vinyl alcohol) hydrogels without crosslinking agents; the freeze–thawing method	-swelled 10-fold their initial weight, and after 20 h, hydrogel samples swelled up to 15-fold; -encapsulation efficiency was equal to 70% in all cases.	-the release of diflunisal from the hydrogels was for 30 h; -higher release profile is for sample CP4-80; -burst effect was not detected for any type of the hydrogels.	[35]
5	70% soya bean lecithin, 30% butyl lactate and 23% water; Lipoid S75 and Phospholipoin 85 G; lipogel form and hydrogel microemulsion;	-skin penetration; -gel has better spreadability and demonstrates a 5.07-fold increase in the transdermal flux as it was compared to Carbomer^®^ 934 gel; -lipogel LO1 demonstrated the ultimate permeability level (210.8 µg cm^−2^ h^−1^) and advanced percentage diflunisal permeated; -the in vivo antihyperalgesia assay showed significant reduction of the licking time in the treated group compared to the control group.	−	[36,37]
6	pH-Sensitive hydrogels based on bovine serum albumin hydrophilic microspheres	−	-release profile depends on diflunisal–polymer matrix interacting and diffusional restriction related to degree of crosslinking in the microparticles; -at pH 6.8, the diflunisal released amount increased (*w*/*w* > 75% after 24 h).	[38]
7	β-Cyclodextrin (βCD), γ-cyclodextrin (γCD), and hydroxypropyl-β-cyclodextrin (HPβCD)	-hydrophilic polymers (carboxymethyl cellulose sodium, polyvinyl alcohol, and poloxamer-188 (PXM188)) were used, the effect of the polymer addition on the solubility and dissolution has been studied. -better solubility was for βCD and HPβCD inclusion complexes. -maximum of diflunisal solubility (1259.5 ± 0.5 µg/mL) was detected for the complex with hydroxypropyl β-cyclodextrin and Poloxamer-188.	-the diflunisal release from β-cyclodextrin and hydroxypropyl β-cyclodextrin complexes was higher than of pure diflunisal in 11–21 times; -hydrophilic polymers allow increasing the release rate of diflunisal in 15–28 times.	[45,46]
8	Cobalt(III)–polypyridyl complexes	-kill the cancer stem cells and the majority of bulk cancer cells even at low concentrations; -killing mechanism of cancer cells by composition number 5 includes the DNA damage and inhibition of COX-2; -against breast cancer cells HMLER and breast cancer stem cells-enriched HMLER-shEcad; -did not possess any toxicity toward normal skin fibroblast cells (line GM07575).	-differentially release the drug under acidic conditions; -complexes selectively release diflunisal/1,10-phenanthroline-bearing complex 5 displays selective potency toward hard-to-kill cancer stem cells (CSCs) (IC50 = 2.1 ± 0.1 μM) over bulk cancer (IC50 = 3.9 ± 0.2 μM) and normal cells (IC50 = 21.2 ± 1.3 μM). This complex induces CSC apoptosis by DNA damage and cyclooxygenase-2 inhibition.	[51]
9	Copper (II) complexes with O-donor ligand *N*,*N*-dimethylformamide or N-donor heterocyclic ligands (2,2′-bipyridine, 2,2′-bipyridylamine, 1,10-phenanthroline and pyridine)	-good binding ability to bovine and human serum as well as to calf–thymus DNA.	−	[52]
10	Poly(ethylene glycol) (PEG)	−	The better release was for the drug–polymer ratio of 1:7.	[64]
11	Eudragit RS100 and RL100	−	Changes in the release profile, leading to slow and prolonged kinetic profile.	[66]
12	Eudragit RS100, S100, L100, and ethyl cellulose; pH-dependent, time dependent, and combined pH and time-dependent systems.	-the ratio 2:3:1 Eudragit S100/Eudragit RS100/diflunisal is the most successful; -colon-specific delivery for the treatment of a variety of diseases, such as nonspecific ulcerative colitis, cirrhosis disease, intestinal amoebiasis, colon tumor.	The release was 0.22 ± 0.03% of the drug included in it in the stomach pH and 26.29 ± 0.91% of the drug in the intestine pH and 77.59 ± 1.79% of the drug in the colon pH.	[67]
13	Ethylenediamine (EDA) core polyamidoamine (PAMAM) dendrimers	-improve transdermal delivery of diflunisal and its pharmacokinetic and pharmacodynamics profiles; - enhance transdermal delivery of diflunisal; -diflunisal–PAMAM complexes lead to 2.48-fold increase in drug level.	−	[72]

Abbreviated terms: COX-2—cyclooxygenase-2; CS—chitosan; CSC—cancer stem cells; DIF—diflunisal; DNA—deoxyribonucleic acid; EDA—ethylenediamine; FVB/NJ—multipurpose inbred mice strain; GM07575—normal skin fibroblast cells; HMLER—bulk breast cancer cells; HMLER-shEcad—breast cancer stem cells-enriched; HPβCD—hydroxypropyl-β-cyclodextrin; k-CAR—k-carrageenan; Lipogel LO1—organogel based on Lipoid S75; MC3T3-E1—osteoblast precursor cell line derived from musculus (mouse) calvaria; NE—nanoemulsion; PAMAM—polyamidoamine; PEG—poly(ethylene glycol; PXM188—poloxamer-188; SLN gel—solid lipid nanoparticles gel; βCD—β-cyclodextrin; γCD—γ-cyclodextrin.

## Data Availability

Not applicable.
